# Machine-Learning-Assisted Nanozyme-Based Sensor Arrays: Construction, Empowerment, and Applications

**DOI:** 10.3390/bios15060344

**Published:** 2025-05-29

**Authors:** Jinjin Liu, Xinyu Chen, Qiaoqiao Diao, Zheng Tang, Xiangheng Niu

**Affiliations:** 1 School of Public Health, Hengyang Medical School, University of South China, Hengyang 421001, China; 2022000136@usc.edu.cn (J.L.); chenxinyu@stu.usc.edu.cn (X.C.); diaoqiaoqiao@stu.usc.edu.cn (Q.D.); tangzheng@usc.edu.cn (Z.T.); 2 The First Affiliated Hospital, Hengyang Medical School, University of South China, Hengyang 421001, China

**Keywords:** nanozyme, sensor array, machine learning, environmental detection, food analysis, biomedical sensing

## Abstract

In the past decade, nanozymes have been attracting increasing interest in academia due to their stable performance, low cost, and easy modification. With the catalytic signal amplification feature, nanozymes not only find wide use in traditional “lock-and-key” single-target detection but hold great potential in high-throughput multiobjective analysis via fabricating sensor arrays. In particular, the rise of machine learning in recent years has greatly advanced the design, construction, signal processing, and utilization of sensor arrays. The constructive collaboration of nanozymes, sensor arrays, and machine learning is accelerating the development of biochemical sensors. To highlight the emerging field, in this minireview, we created a concise summary of machine-learning-assisted nanozyme-based sensor arrays. First, the construction of nanozyme-involved sensor arrays is introduced from several aspects, including nanozyme materials and activities, sensing variables, and signal outputs. Then, the roles of machine learning in signal treatment, information extraction, and outcome feedback are emphasized. Afterwards, typical applications of machine-learning-assisted nanozyme-involved sensor arrays in environmental detection, food analysis, and biomedical sensing are discussed. Finally, the promise of machine-learning-assisted nanozyme-based sensor arrays in biochemical sensing is highlighted, and some future trends are also pointed out to attract more interest and effort to promote the emerging field for better practical use.

## 1. Introduction

Since Fe_3_O_4_ nanoparticles (NPs) were observed to present peroxidase (POD)-like catalytic activity to trigger several chromogenic reactions in 2007 [1], nanoscale materials with enzyme-mimetic features (nanozymes) have attracted great attention in academia [2,3,4]. Compared with natural enzymes, nanozymes display several attractive merits, including stable performance under complex conditions, easy mass production, and low cost [5]. Furthermore, their catalytic properties can be flexibly tailored according to demands. With these advantages, they have found great application promise in analytical sensing [6], environmental engineering [7], food processing [8], and biomedicine [9,10,11]. Especially in the analytical sensing area, the catalytic signal amplification feature enables nanozymes as labels in enzymatic cascade reactions, aptasensors, and immunoassays to provide sensitive responses [12,13,14]. In addition, many nanozyme materials exhibit specific interactions with some targets, endowing them with extensive use in selectively detecting environmental pollutants [15], food hazard factors [16], and biochemical species [17] in a “lock-and-key” paradigm.

In real samples, multiple species with similar structures or/and properties often co-exist. It is hard to recognize a single target of interest in a complex environment. Additionally, sometimes it is desired to detect multiple targets simultaneously. For these cases, sensor arrays can play an important role. Inspired by the human olfactory and gustatory systems, sensor arrays are composed of multiple sensing units. Through cross-reacting with multiple targets, they can offer discrepant responses for each species [18]. These responses are converted into measurable signals including optical and electrical readouts. With the help of information processing methods such as pattern recognition [19], they can be utilized to classify, identify, and even quantify the analytes synchronously, just like a human tongue or nose that is able to distinguish different tastes or smells [20]. Hence, sensor arrays have been intensively explored for high-throughput detection and complex sample analysis. In comparison with traditional “lock-and-key” biosensors, sensor arrays significantly expand detection range and improve analytical efficiency. Sensing units are the core of a sensor array. Among various sensing units, nanozymes have drawn increasing attention in the past few years because of their abundant materials, activities, and signal readouts. By utilizing the nonspecific interactions between nanozymes and targets as well as their impacts on nanozyme catalysis, unique fingerprints for the targets can be collected for qualitative and quantitative detection [21,22].

In the above sensor arrays, an important process is to first extract useful information from the complex and disordered signals generated and then perform information processing to output accurate results. This process requires the use of machine learning. In recent years, the applications of machine learning in chemical analysis and sensors have become a hotspot in research. With the potential of mining hidden information and processing complex signals rapidly, machine learning is playing a significant role in biochemical sensing [23]. Machine learning algorithms can quickly conduct in-depth analysis of large-scale data through intelligent processing, thus providing high efficiency and good accuracy. For sensor arrays, they require a cluster of processes to treat multidimensional data matrices with no prominent categorical feature. Traditional dimensionality reduction, classification, and clustering techniques, mainly including linear discriminant analysis (LDA), principal component analysis (PCA), and hierarchical cluster analysis (HCA), have been widely applied in sensor arrays to mine the internal characteristics and relationships. Furthermore, advanced machine learning algorithms such as support vector machines (SVMs), linear/nonlinear regressions, artificial neural networks (ANNs), and random forest (RF) have also been utilized in sensor arrays to classify and predict results.

**Figure 1:**
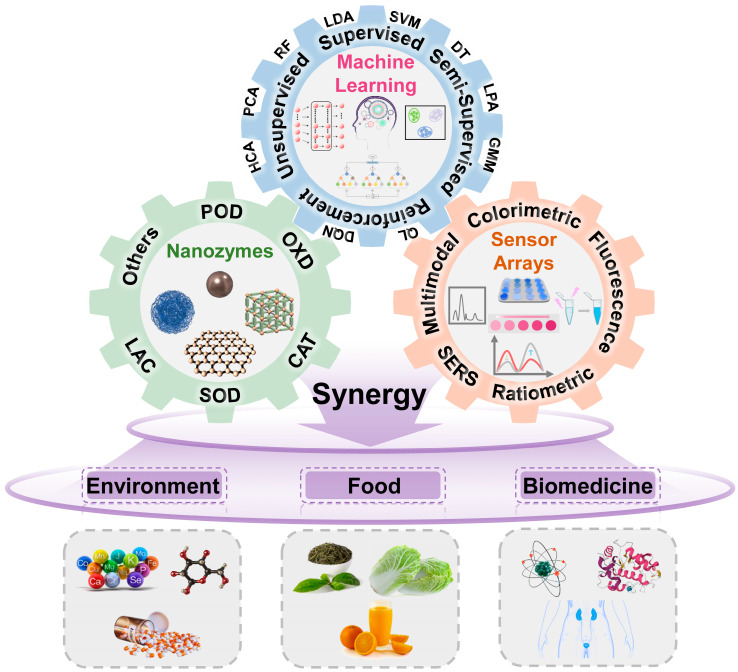
Scheme of machine-learning-assisted nanozyme-based sensor arrays for environmental detection, food analysis, and biomedical sensing.

According to the above introduction, it is not difficult to see that the constructive collaboration of nanozymes, sensor arrays, and machine learning can realize the multichannel screening and detection of multiple targets in complex matrices with precise results. Nanozymes provide sensitive responses; sensor arrays offer complex, intersectional signals; and machine learning helps process these signals to give accurate results. In fact, machine-learning-assisted nanozyme-based sensor arrays are undergoing a rapid development period. To highlight the up-to-date progress achieved in the emerging field, in this minireview, we summarize machine-learning-assisted nanozyme-based sensor arrays from the construction, empowerment, and application aspects. Unlike in previous publications related to nanozyme-based sensor arrays [21,22], where nanozyme materials and activities as well as their use were highlighted (Table 1), in this work, we first introduce the fabrication of nanozyme-involved sensor arrays, with emphasis on sensing variables and signal outputs. We propose eight major sensing variables, including material difference, environmental control, temporal kinetics, and multiwavelength, multisubstrate, and multidimensional signal intercrossing, to form a “variable combination library”. These variables are integrated with monomodal signals such as colorimetry, fluorescence, and photothermal effects to establish multimodal arrays. Furthermore, the critical role of machine learning in signal processing, information extraction, and result feedback is emphasized. This led to the development of a complete technological framework (Figure 1): “nanozyme activity regulation–multivariable design of sensor arrays–full-process empowerment by machine learning”. After that, some typical applications of machine-learning-assisted nanozyme-involved sensor arrays in environmental detection, food analysis, and biomedical sensing are discussed. Finally, the great potential of machine-learning-assisted nanozyme-based sensor arrays in biochemical sensing is anticipated, and some development trends are presented. It is believed that our minireview can provide a useful reference to the emerging field, and we also hope to attract more research effort to promote machine-learning-assisted nanozyme-based sensor arrays from scientific research to practical use.

## 2. Construction of Nanozyme-Based Sensor Arrays

### 2.1. Nanozyme Materials and Activities

Nanozymes present several attractive merits over bioenzymes. First, the synthesis of nanozymes is relatively simple, and their cost is controllable. Second, nanozymes are more stable under complicated conditions, benefiting their wider practical application. Third, nanozymes can better adapt to working environments with wider pH and temperature ranges. Fourth, it is easier to adjust their catalytic features vis surface modification, component doping, size control, and so on. These characteristics give them great potential in the biochemical sensing field.

From the material aspect, various material types have been explored as artificial enzyme mimics, mainly including transition metal oxides/sulfides/hydroxides [24], noble metals [25], carbon-based materials [26], and metal–organic frameworks (MOFs) [27]. For instance, Fe-based oxides and single-atom materials can present POD, oxidase (OXD), catalase (CAT), and superoxide dismutase (SOD) activities [28]; Au NPs are reported to exhibit glucose oxidase (GOx)- and POD-like activities for fabricating cascade systems [29]; and functionalized carbon can show several oxidoreductase activities [30]. As the nanozyme materials used to fabricate sensor arrays were summarized and discussed in a previous review [22], here, we do not enter into a deep discussion, and one can refer to said publication for further information.

From the perspective of catalytic activity, a majority of current research focuses on the oxidoreductase type, mainly including POD, OXD, CAT, SOD, and laccase (LAC). These activities can further be roughly divided into antioxidation and pro-oxidation. In general, antioxidation nanozymes include SOD, CAT, glutathione peroxidase, and thioredoxin peroxidase. They can effectively remove reactive oxygen species (ROS) from living organisms to avoid toxic reactions. Pro-oxidation nanozymes contain OXD, POD, GOx and so on. They can catalyze H_2_O_2_ or dissolved O_2_ and promote substrate oxidation to provide colorimetric, fluorescence, electrochemical, or/and Raman signals. Apart from the redox-type nanozymes, a few hydrolase-like nanozymes have also been developed in recent years for catalytic applications [31,32,33].

### 2.2. Sensing Variables

**Figure 2:**
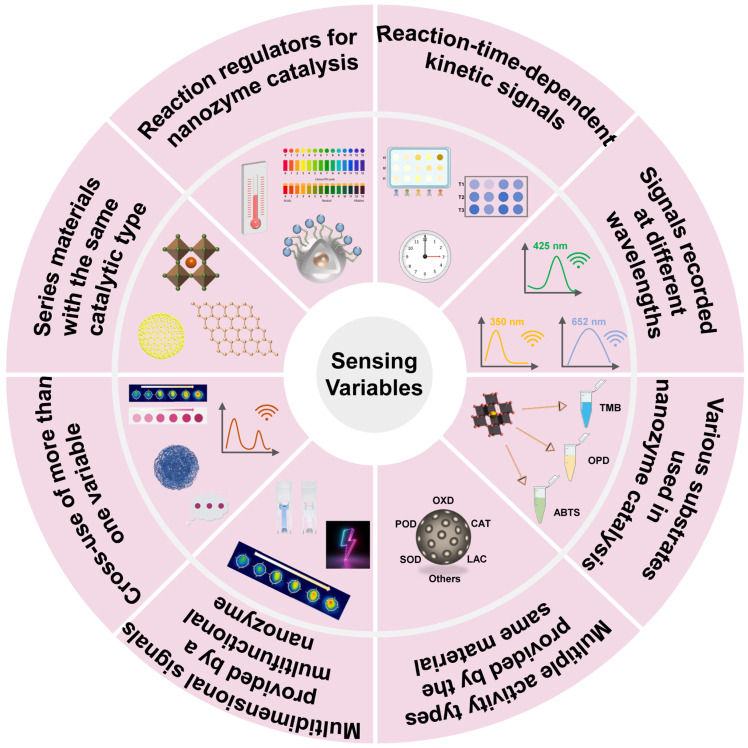
Various sensing variables that can be used to fabricate nanozyme-involved sensor arrays.

To construct a high-performance and applicative sensor array, it is very important to select an appropriate sensing variable that can not only provide distinguishable cross-signals for similar targets but simplify the detection operation and signal treatment processes as much as possible. To this end, nanozymes provide a variety of variables for the construction of sensor arrays, as summarized in Figure 2.

First, one can synthesize a series of nanozyme materials with the same catalytic type and employ their discrepant activities and interactions with the targets to obtain unique fingerprints for each target [34,35,36,37,38,39,40,41]. For instance, Wei’s group prepared graphene oxide (GO), nitrogen-doped graphene (NG), and nitrogen–sulfur codoped graphene (NSG) to fabricate a sensor array for pesticides [39]. All three materials showed the POD-like catalytic feature and could trigger the oxidation reaction of 3,3′,5,5′-tetramethylbenzidine (TMB) with H_2_O_2_ to form a recognizable blue color. Although they had the same catalytic type, these POD nanozymes presented different abilities to induce the chromogenic reaction to varying degrees. When aromatic pesticides were present, the active sites of these nanozymes were covered through nonspecific adsorption, resulting in different degrees of reduction in their POD performance and thus different changes in the color signal. According to this principle, the developed sensor array based on the three nanozyme materials could identify five pesticides (lactofen, bensulfuron-methyl, fluoroxypyr-meptyl, diafenthiuron, and fomesafen) and their various mixtures accurately. Such a sensing variable depends on the use of different nanozyme materials, which increases the material cost in fabricating a sensor array.

Second, as nanozyme catalytic reactions usually occur under preferable conditions, and their kinetics are obviously affected by the reaction environment applied, some simple regulators, such as buffer pH [42,43,44,45] and additional substances [46,47], can be employed as variables to constructure nanozyme-involved sensor arrays. Niu’s group constructed a three-channel colorimetric sensor array using Au_2_Pt NPs with good POD-like activity [42]. Interestingly, the color reaction between TMB and H_2_O_2_ catalyzed by Au_2_Pt NPs was highly sensitive to the buffer pH value. When tracking the color development process in three buffer solutions with different pH values (pH = 3.5, 4.0, and 4.5), different amounts of blue oxTMB were generated and recorded. As different antioxidants (vitamin C, glutathione, caffeic acid, gallic acid, and cysteine) could reduce the oxTMB product again and cause the fading of the blue color to various degrees, accurate identification of the five antioxidants at different levels as well as their mixtures was achieved. As for regulation by additional substances, Bi’s group designed a cross-reaction colorimetric sensor array based on terephthalic-acid-modified graphene quantum dots (TPA@GQDs) with metal-ion-regulated enzyme-like activity [46]. The TPA@GQDs, as a nanozyme with inherent POD activity, could catalyze H_2_O_2_ to form hydroxyl radicals, which then oxidized the TMB substrate to produce an ultraviolet–visible (UV-vis) absorption signal at the maximum wavelength of 650 nm. As the chosen transition metal ions (Zn^2+^, Cu^2+^, and Fe^2+^) had different Fenton activities, they could cooperate with the rich hydroxyl group on the surface of the TPA@GQDs to induce the generation of new catalytic sites, and consequently, the POD-mimicking activity of TPA@GQDs could be improved by the three metal ions to different extents. When mercaptans were added, the stronger binding between the sulfhydryl group in the mercaptans and the transition metal ions made the analytes preferentially bind to these ions, thus leading to the catalytic performance inhibition of metal ion upregulated TPA@GQDs. As a result, different absorbance changes were generated for each mercaptan. With the established sensor array, different types and concentrations of mercaptans as well as their mixtures at different proportions were identified accurately. Compared with the first category, where different nanozyme materials are required, the use of simple regulators can reduce material costs. However, current applicable regulators that can provide unique fingerprints for multiple targets in complex samples are very limited.

Third, given that nanozyme catalytic reactions exhibit typical enzymatic kinetics, the signals generated at different time points can be captured and used for constructing nanozyme-involved sensor arrays [48,49]. Recently, our group designed a facile single-nanozyme single-signal sensor array for the identification and brewing evaluation of Chinese tea [49]. In detail, manganese oxyhydroxide (MnOOH) nanowires were employed as the only sensing material; these exhibited good OXD-like activity to catalyze the colorless TMB oxidation with dissolved O_2_ to blue oxTMB. In fact, the oxidation process was time-dependent. When the reaction kinetics were monitored upon reaction time, the absorbance of oxTMB recorded was different. Tea polyphenols with abundant hydroxyl groups presented reducibility to inhibit the color development of TMB, and the degrees to which the targets inhibiting TMB oxidation were highly dependent on their different reducing abilities. Consequently, three reaction times were chosen according to Euclidean distance, and a data pattern with three reaction times×six targets×six repeats was constructed by combining HCA and LDA to identify polyphenols in Chinese tea. The developed array could not only effectively differentiate a single target at different levels, as well as their various mixtures, but successfully identify the influence of different brewing methods. Comparatively speaking, the time-resolved, kinetics-dependent sensing variable was simple in operation and effective in cost, well suitable for building simple sensor arrays based on nanozyme catalysis.

Fourth, the absorbance values recorded at different wavelengths from the same enzymatic product also indicate the characteristics of similar targets, which can be utilized to fabricate sensor arrays for identification [50,51,52]. For example, Bai et al. combined acetylcholinesterase (AChE) and fullerene@MOF to construct an efficient enzyme–nanozyme cascade continuous flow reactor for the detection of multiple organophosphorus (OPs) [50]. In their prepared nanozyme, fullerene was encapsulated in MOF-545-Fe, and its unique host–guest interaction maximized both the OXD and POD activities. Considering the inhibiting effect of OPs on the activity of AChE, the enzyme–nanozyme cascade continuous flow reactor was fabricated by integrating AChE with fullerene@MOF-545-Fe through supramolecular interactions. According to the absorbance difference of the oxTMB product generated at three wavelengths (371, 568, and 652 nm), a colorimetric array with three channels was designed to achieve the sensitive detection and identification of glyphosate, omethoate, and paraoxon.

Fifth, different substrates can be applied as electron donors to take part in nanozyme reactions, providing discrepant responses to similar analytes for their identification [53,54,55]. A typical example comes from Dai’s group [53], where they constructed a cross-reactive and cost-effective nanozyme-involved sensor array of OPs. In their study, Ag_2_O nanospheres with simulated OXD activity were employed as the sensing unit, and they could catalyze the oxidation of TMB, *o*-phenylenediamine (OPD), and 2,2′-azino-bis(3-ethylbenzthiazoline-6-sulfonic acid) (ABTS) to result in different color development reactions. Because of the differences among the three substrates in electronegativity, reaction affinities, and molar extinction coefficients, they could be catalytically oxidized to provide varying absorbance values. As a result, colorimetric analysis of each analyte was performed based on the fact that the presence of OPs directly suppressed the catalytic activity of Ag_2_O nanospheres toward the three substrates.

Sixth, different catalytic types are often observed in the same material [56]. When these activities are employed to catalyze corresponding reactions, cross fingerprints can be collected to fabricate sensor arrays for multiple target analysis [57,58]. For instance, Chen et al. established a colorimetric sensing array based on an ultrasmall CuMn–histidine complex, which exhibited favorable POD-like activity at neutral pH due to the electrostatic effect and hydrogen bonding [57]. Interestingly, the material also presented LAC and SOD activities at the same pH value. By using the three activities to catalyze corresponding reactions, a nanozyme-involved color sensor array was fabricated and validated for the distinguishment of biothiols. The method, while effective in identifying multiple targets in complex matrices, requires the use of different reaction systems and the record of corresponding signals, both of which make the developed sensor array cumbersome in operation.

Seventh, apart from the enzyme-mimetic catalytic feature, many nanozymes also tend to present electrical, optical, magnetic, and thermal properties [59]. Combining the nanozyme catalytic feature and these properties in the same material is considered to enable some desirable advantages in biochemical analysis [60]. With multifunctional nanozymes, signals from different modes and dimensions can be recorded to provide cross-reaction signals for array sensing [61,62]. Recently, Li’s group elaborately utilized the photothermal signal of a nanozyme (L-aspartic acid-Cu) as one of the sensing units and constructed a multisignal sensor array integrating the colorimetric, fluorescence, and photothermal responses together [61]. In their system, the POD nanozyme was utilized to catalyze the colorless TMB substrate into blue oxTMB. Meanwhile, the fluorescence of TMB gradually decreased during the process. The generated oxTMB showed the photothermal effect under 808 nm irradiation, resulting in an increase in the reaction system temperature. By recording the oxTMB blue color, the TMB intrinsic fluorescence, and the system temperature originating from the oxTMB photothermal effect, the fabricated array combing LDA and HCA could detect and identify sulfonylurea pesticides from 0.1 to 100 μg/mL, and the correct recognition rate reached 100%. Furthermore, by integrating the array with the K-nearest neighbor (KNN) algorithm, a concentration-independent model was further established to identify these targets with 100% accuracy. More sensor arrays are expected to be developed according to this idea, with multidimensional signals when using multifunctional nanozymes (fluorescent nanozyme, electroactive nanozymes, and so on).

Last but not least, all the sensing variables mentioned above can be cross-used to provide more precise results for array sensing [63,64,65,66,67,68,69]. For instance, Ren et al. prepared a Fe, Se codoped carbon material with OXD activity to fabricate a sensor array of sulfur-containing compounds, where four fingerprint extraction signals were obtained at two buffer pH values (pH = 3.0 and 4.0) and two absorption wavelengths (λ = 370 and 652 nm) of the oxTMB enzymatic product [69]. As a result, nine sulfur-containing compounds based on different inhibiting effects were well identified by the developed multichannel colorimetric sensor array combining PCA and HCA. Nanozymes and the corresponding catalytic reactions offer rich sensing units and parameters, and the cross-use of these units and parameters can lead to huge combinations of variables to fabricate sensor arrays. Table 2 summarizes the main features of the sensing variables available in fabricating nanozyme-involved sensor arrays.

### 2.3. Signal Outputs

In nanozyme catalysis, a few substrates (TMB, ABTS, OPD, diaminobenzidine, luminol, and terephthalic acid) are often employed to generate colorimetric, fluorescence, electrochemical, chemiluminescence, or/and Raman responses [27]. Furthermore, some substrates or products can provide more than one signal. Taking TMB enzymatic oxidation as an example, colorless TMB can be oxidized into oxTMB with a blue color, which finally becomes yellow after acid treatment, offering colorimetric signals for sensing; the oxTMB product with a biphenyl structure can quench additional fluorescent agents for fluorescence detection; the TMB substrate shows an intrinsic weak fluorescence, while its oxidation product oxTMB does not have this feature; unlike TMB, the oxTMB product can provide characteristic Raman signals for surface enhanced Raman scattering (SERS) detection; and photothermal and photoacoustic effects are found in the TMB catalytic oxidation system [70]. All of these responses offer optional signal readouts for array sensing.

Colorimetry is a common analytical technique employing the UV-vis absorption properties of colored substances at specific wavelengths. Oxidation of chromogenic substrates in the presence of nanozymes produces characteristic colors, and different analytes can be distinguished according to color shading. For colorimetric analysis in nanozyme catalysis, the commonly used substrates include TMB, ABTS, OPD, and diaminobenzidine (DAB). The colorimetric analytic method is relatively simple in operation and instrument, and it can sometimes be completed even by the naked eye. Furthermore, the colorimetric signals can be recorded, analyzed, and fed back by some personal smart appliances such as smartphones [34,36]. With these features, colorimetric signals are often captured in nanozyme-involved sensor arrays for multiple target analysis [37,38,55,63,65,71,72].

In general, fluorescence sensor arrays are considered to be superior to colorimetric ones in terms of detection sensitivity. To this end, some researchers have employed fluorescence from nanozyme catalysis as an output signal to build sensor arrays [73,74]. For instance, in Chen’s group, three-dimensional fluorescence spectra generated by the interactions of OXD-like CuO NPs with sugars were employed as the input data sets [74]. The three-dimensional fluorescence spectra were collected at excitation and emission wavelengths as many as possible. The established fluorescence intensity dataset was analyzed by an optimized convolutional neural network (CNN) algorithm to separate the selected sugars via capturing subtle structural differences.

The majority of nanozyme sensing systems rely on single-signal output. However, these systems are susceptible to environmental and personal factors. By comparison, analytical platforms featuring ratiometric outputs can provide more convincing and stable performance in sensor arrays [75,76,77]. Wei’s group developed a proportional fluorescence sensing method using fluorescent nanozymes [75]. The proposed three C_3_N_4_-based nanozymes (C_3_N_4_−Cu, C_3_N_4_−Ru, and C_3_N_4_−hemin) exhibited an intrinsic fluorescence peak at 438 nm when they were excited at the wavelength of 385 nm. When both OPD and H_2_O_2_ were in presence, the C_3_N_4_-based nanozymes could catalyze the oxidation of colorless OPD to produce yellow oxOPD, which emitted fluorescence at 564 nm and at the same time quenched the 438 nm fluorescence of the nanozymes. As the fluorescence ratio (F_564_/F_438_) increased along with H_2_O_2_ concentration, a ratiometric fluorescence method was established and verified for the array sensing of five phosphates.

Certain nanozymes containing noble metals can offer SERS capacity, which enables the expedient recording of the Raman signal of the SERS-active reporter TMBox generated from nanozyme catalytic reactions. In this regard, Wang et al. found that certain Ag-containing materials possessed both OXD-mimicking and SERS features [78]. They established a multichannel SERS paper-based sensor array using three Ag-based nanozymes for the quantification and identification of six biothiols. In their study, Ag/Mn_3_O_4_, Ag_3_PO_4_, and silver citrate (Ag_3_Cit) were loaded on Whatman filter papers to fabricate SERS paper chips with three sensing channels. In the presence of biothiols, each SERS sensing channel could generate different oxTMB signal variations via the differences in the inhibition ability of diverse biothiols and the exclusive property of each Ag-based material. After the SERS signal variations were plotted as specific fingerprints of each target and further translated into intuitive clustering profiles through LDA and HCA, six biothiols could be precisely identified with minimum concentrations as low as 1 μM.

Compared with single-mode detection, multimodal analytical sensing can significantly improve comprehensive detection capability by integrating multiple signals (optical, electrochemical, thermal, acoustic, etc.) together in a sensor array. Nanozymes can synchronously generate optical, electrochemical, thermal, and other multimodal signals through catalyzing reactions, significantly improving the detection sensitivity and reliability. Multimodal cross-validation can reduce false positive/negative results and adapt to the interference environment of complex samples. Nanozymes have also been used to generate multimode signals for array sensing [61,66,79].

## 3. Machine Learning Advances Nanozyme-Based Sensor Arrays

### 3.1. PCA, LDA, and HCA for Target Classification and Clustering

As mentioned above, one of the most important processes in sensor arrays is to extract useful information from complex signals and perform information treatment to output accurate results, where appropriate ML algorithms are essential. PCA, LDA, and HCA are three classical data dimensionality reduction and pattern recognition methods that have been widely used in the data processing of sensor arrays [35,37,38,46,77]. By extracting the principal components in the maximum variance direction, PCA projects high-dimensional sensing data (such as the multi-index response of a gas sensor array) into a low-dimensional space to remove redundant information and highlight core features; this is often used for sensor signal denoising and feature compression. LDA focuses on maximizing interclass differences and minimizing intraclass differences, and it is suitable for labeled data classification (such as distinguishing different odors or pollutant types) and improving classification accuracy by constructing discriminant feature subspaces. According to measurements of similarity (such as Euclidian distance), HCA performs hierarchical clustering of unlabeled sensor responses to visually display the internal structures of the collected data.

In sensor arrays, the combination of the three algorithms is usually used to enable efficient data analysis [35,37,38,46,77]: LDA optimizes the performance of classification models after PCA preprocessing reduces the dimensions, while HCA assists in exploring unknown data patterns, providing a lightweight, highly robust analytical framework for multisensor fusion systems. For instance, Zhang et al. established a colorimetric sensor array of dental caries bacteria based on the OXD catalytic activity of Fe-N-C single-atom nanozymes that could be upregulated or downregulated by the targets to different extents [37]. Machine learning was utilized to assess the colorimetric patterns of various cariogenic bacteria. The analysis using LDA provided unique fingerprints for each caries-causing bacterium. At the same time, HCA was used to evaluate the sensor array’s ability to detect the commonalities among several caries-causing bacteria. With the aid of machine learning, the developed colorimetric sensor array could accurately distinguish ten kinds of cariogenic bacteria, showing broad application prospects in the prevention and treatment of oral diseases.

### 3.2. Other Machine Learning Algorithms for Target Analysis

Apart from PCA, LDA, and HCA, some other advanced machine learning algorithms have also been developed to combine nanozyme-based sensor arrays for multiple target qualitative analysis [47,61,63,65,66,73,80]. For instance, Xu et al. synthesized a multifunctional Cu-1,3,5-benzenetricarboxylic acid (Cu-BTC) material via the coprecipitation method for the intelligent recognition of antioxidant phenolic compounds (APs) with the assistance of machine learning [66]. Thanks to the outstanding LAC-like performance of Cu-BTC, it catalyzed the oxidation of various APs to form colored quinone imines. Furthermore, the nanozyme presented a POD-mimicking catalytic feature to induce the oxidation of colorless TMB to blue oxTMB as well as photothermal properties under near-infrared laser irradiation. Consequently, a bimodal colorimetric/photothermal sensing platform was developed and employed for the discrimination detection of multiple APs. Through combining an ANN algorithm with the sensor array, precise identification and accurate prediction of ten APs in several real samples (black tea, coffee, and wine) were accomplished. Furthermore, the smartphone sensing technique was incorporated into the array to achieve the portable, facile identification of APs in food matrices.

As for quantitative determination, linear/nonlinear regressions are usually achieved via support vector regression (SVR) and RF for content prediction in sensor arrays [34,36,50,55,64,67,71,72,74,81,82]. For example, Zhang et al. constructed a dual-mode probe based on NH_2_-MIL-88B(Fe, Ni) for the colorimetric and fluorescence detection of tetracyclines (TCs) [81]. The NH_2_-MIL-88B(Fe, Ni) with POD-like activity also showed blue light emission. TCs, acting as inhibitors of nanozyme catalytic activity, reduced the UV-vis response of oxTMB. In addition, the targets could quench the blue fluorescence of the nanozyme via an internal filtration effect. Thus, concentration-dependent fluorescence images of TCs could be gained under stimulation. The images were treated by a WeChat applet, and the color information, including RGB values, was extracted by a YOLO v3 algorithm to present the detection results of TC levels. In another example, Li’s group combined polyphenol oxidase and two nanozymes with PPO-mimetic activity together to establish a sensing platform to realize the identification of TPs and Chinese tea [71]. The polyphenol oxidase and the two nanozymes were able to oxidize TPs to interact with 4-aminoantipyrine, which could generate various colors according to the types of TPs. As a result, a color sensing array was developed according to the color differences recorded in the presence or absence of these analytes to identify TPs. The collected array data were treated by LDA, partial least squares discriminant analysis (PLS-DA), and HCA to report the sensing results of six TPs. Moreover, a machine-learning-based dual output model of TP classification and concentration analysis was developed, which could achieve the differentiation of TPs as well as predicting concentrations. The fabrication of such a dual output model also allowed the differentiation of tea series and species.

Despite these recent advancements in machine-learning-assisted nanozyme-based sensor arrays, model selection, dataset challenges, and interpretability, as well as the application of state-of-the-art machine learning methods, remain underexplored. Given the potential of these aspects in enhancing the performance and capabilities of sensor arrays, it is necessary to conduct more in-depth research on their applicable scopes and application scenarios.

First of all, regarding the selection of machine learning models, factors such as the characteristics of data, task requirements, and computing resources need to be comprehensively considered. (1) Data characteristics: if the data are linearly separable, PCA and LDA might be better choices; if the data are nonlinear, SVM and ANN might be more appropriate. (2) Task requirements: for classification tasks, SVM and RF are more effective; for the dimension reduction task, PCA and LDA are more applicable. (3) Computing resources: for large-scale datasets, ANN and RF have higher computational complexity, while PCA and HCA may be more suitable for smaller datasets (Table 3).

Next, when applying machine learning to sensor arrays, the challenge of the dataset is a key factor affecting the performance and generalization ability of the model used. For instance, the sample sizes of different classes or targets in sensor array data vary significantly, possibly leading to data imbalance. Moreover, sensor datasets often contain noise and outliers, which can affect the accuracy and robustness of the model. Additionally, sensor arrays typically generate high-dimensional data, which can increase computational complexity and the risk of overfitting. The above-mentioned problems cannot be ignored. To effectively address issues such as imbalance, noise, and drift in real-world sensor data, the following comprehensive machine learning strategies can be designed: (1) removing noise and outliers through data preprocessing methods (such as filtering, smoothing and outlier detection) to improve data quality; (2) utilizing data augmentation techniques (such as rotation, scaling, adding noise, etc.) to increase data diversity and enhance the robustness of the model against noise and drift; (3) adopting feature selection and feature extraction methods (such as PCA, statistical-based methods) to reduce the data dimension, decrease the risk of overfitting, and improve the interpretability of the model at the same time; (4) selecting a model suitable for the characteristics of the data and combining model integration methods (such as random forest, gradient hoist, etc.) to enhance the generalization ability of the model; (5) using regularization techniques (such as L1 and L2 regularization) to constrain the model complexity, prevent overfitting, and evaluate the generalization performance of the model through cross-validation. Through the comprehensive application of these strategies, the performance and reliability of machine learning models when dealing with complex sensor data can be significantly improved, thereby better addressing challenges such as noise, imbalance, and drift.

The interpretability of machine learning models refers to the ability to understand and explain how a model arrives at its predictions. As machine learning has been increasingly applied in nanozyme-based sensor arrays, the importance of interpretability has become more pronounced. Interpretability tools, such as Shapley additive explanations (SHAP) and local interpretable model-agnostic explained (LIME), have been used to clarify the decision-making process of complex models. The SHAP value originates from “game theory”. By evaluating the average marginal contribution of each feature in all possible alliances, it provides a method to quantify the contribution of each feature to the model prediction. This method is helpful for understanding which features are the most influential when driving model decisions. For example, in a sensor array, the SHAP value can identify which specific sensor responses or environmental conditions have the greatest impact on the model’s output, thereby providing insights into the underlying mechanisms of sensor behavior. Similarly, LIME aims to explain individual predictions by locally approximating complex models using interpretable models such as linear regression. By interfering with the input data and observing the changes in the model predictions, LIME can identify the key features that contribute to specific predictions. This local interpretability is particularly useful when understanding the model behavior of a specific instance, which is crucial for identifying anomalies or verifying the model’s decisions in practical applications. However, despite these advancements in interpretability, there are still some challenges. First, the complexity of high-dimensional data in sensor arrays based on nanozymes makes it difficult for interpretability tools to provide clear and concise explanations. A large number of features and their potential interactions may mask the most relevant factors, leading to incomplete or misleading explanations. Second, the computational cost of these interpretability methods can be very high, especially when dealing with large datasets or multilayer models. This might limit their practical application in real-time or resource-constrained environments. Addressing these challenges requires continuous research and development of more powerful and effective interpretable technologies, as well as a deeper understanding of the specific requirements and limitations of nanozyme sensor applications.

Finally, although the most advanced deep learning methods are currently less applied in nanozyme-based sensor arrays, these methods have significant potential and can provide new ideas for the performance improvement and application expansion of nanozyme-based sensor arrays. For example, CNNs perform well in processing image and sequence data and are capable of automatically extracting local features and capturing the spatial hierarchy in data. In nanozyme-based sensor arrays, CNNs can be used to process the image data generated, and they can identify and quantitatively determine targets by analyzing the optical response images of sensor arrays. Furthermore, CNNs can also be used to process the time series data of sensor arrays to enhance the detection capability for dynamic changes. However, CNNs also face problems such as insufficient training and poor generalization ability caused by small datasets. Meanwhile, their complex model structure is prone to overfitting, and they have a high demand for computing resources along with poor interpretability. Furthermore, unbalanced data distribution may also cause the model to be biased towards the majority class, affecting the detection performance of the minority class. GNNs are particularly suitable for processing data with complex relationships, such as nodes and edges in sensor networks. In nanozyme-based sensor arrays, GNNs can be used to model the interactions among sensors, thereby better understanding the overall response mechanism. By regarding each sensor in nanozyme-based sensor arrays as a node in graphs, a GNN can capture the interrelationships among the sensors and utilize these relationships to enhance the detection performance for complex samples. However, the application of GNNs also faces many challenges. First of all, the performance of a GNN highly depends on the quality and accuracy of the graph structure. If the construction of the graph is inaccurate, for example, the connection relationship between nodes is incorrect or missing, this may cause the model to learn incorrect patterns, thereby affecting the reliability of the detection results. Second, the training and inference processes of GNNs usually require a large amount of computing resources, especially when dealing with large-scale graph data, which may limit their application in resource-constrained scenarios. In addition, similarly to CNNs, GNNs are prone to overfitting problems, especially when the amount of data is limited. The model may overfit the training data and fail to generalize new and unseen data. Finally, the interpretability of GNNs is still limited. Although they can capture the relationships between nodes, their internal decision-making process is still difficult to understand intuitively. Attention-based models (AMs) can automatically focus on the most informative parts of data, thereby improving the interpretability and performance of the model used. In nanozyme-based sensor arrays, the attention mechanism can be used to identify the features in sensor arrays that are most sensitive to targets. By applying the attention mechanism to the feature extraction process of sensor arrays, the model can automatically identify which sensor response is the most critical for the detection of multiple targets. However, the performance of AM is highly dependent on the quality of the input data and the distinguishability of the features. If there is a lack of clear “focus points” in the data, an AM may not function effectively. In addition, AMs usually need to be used in combination with other models (such as CNNs or GNNs), which may lead to an increase in the overall model complexity and an extension of training and inference time. In the case of limited data volume, AM may also have the problem of overfitting. Although the application of CNNs, GNNs, and AMs in nanozyme-based sensor arrays is still in its infancy, the potential of these methods is huge. They can provide new ideas and tools for improving the performance and expanding the application of nanozyme-based sensor arrays.

## 4. Applications of Machine-Learning-Assisted Nanozyme-Based Sensor Arrays

With the functions and characteristics mentioned above, machine-learning-assisted nanozyme-based sensor arrays hold great potential in the fields of environmental detection and food analysis as well as biomedical sensing. Table 4 lists some representative examples regarding the nanozyme material, activity, sensing variable, signal mode, and machine learning algorithm of sensor arrays and their analytical applications.

### 4.1. Environmental Detection

Currently, some machine-learning-assisted nanozyme-involved sensor arrays have been explored to analyze multiple metal ions [52], inorganic nonmetallic ions [83], phenolic pollutants [84,85], and antibiotics [80,81]. For metal ions, Zhang’s group fabricated a nanozyme-involved sensing array using a single nanozyme (hollow carbon nanospheres with loading of Pt NPs, Pt/HCNs) with three colorimetric channels to achieve the analysis of different metal ions [52]. Adsorption peaks at 370, 450, and 650 nm were recorded and attributed to the catalytic oxidation of TMB by Pt/HCNs with OXD activity. When metal ions (Pb^2+^, Fe^3+^, Zr^4+^, Mg^2+^, Cr^3+^, Ag^+^, and Hg^2+^) were added to the Pt/HCNs + TMB system, the three absorption peaks presented different responses. After the data were processed by dimensionality reduction using LDA, rapid, facile, and effective analysis of the seven metal ions in the environment was achieved.

For phenolic pollutants, our group reported a new sensor array for the colorimetric quantification and discrimination of phenolic pollutants by using Fe_3_O_4_ NPs as a POD mimic [84]. In the study, small-sized Fe_3_O_4_ NPs were prepared and employed to catalyze the oxidative coupling of phenolic substances and 4-aminoantipyrine in the presence of H_2_O_2_, presenting an obvious color change to suggest target concentration. Furthermore, a detection array was established according to the different reaction kinetics to identify multiple phenolic compounds. When integrating the sensor array with PCA, six common phenolic pollutants (phenol, *o*-chlorophenol, *m*-chlorophenol, *p*-chlorophenol, *m*-aminophenol, and *o*-nitrophenol) could be well differentiated even at low concentrations.

**Figure 3:**
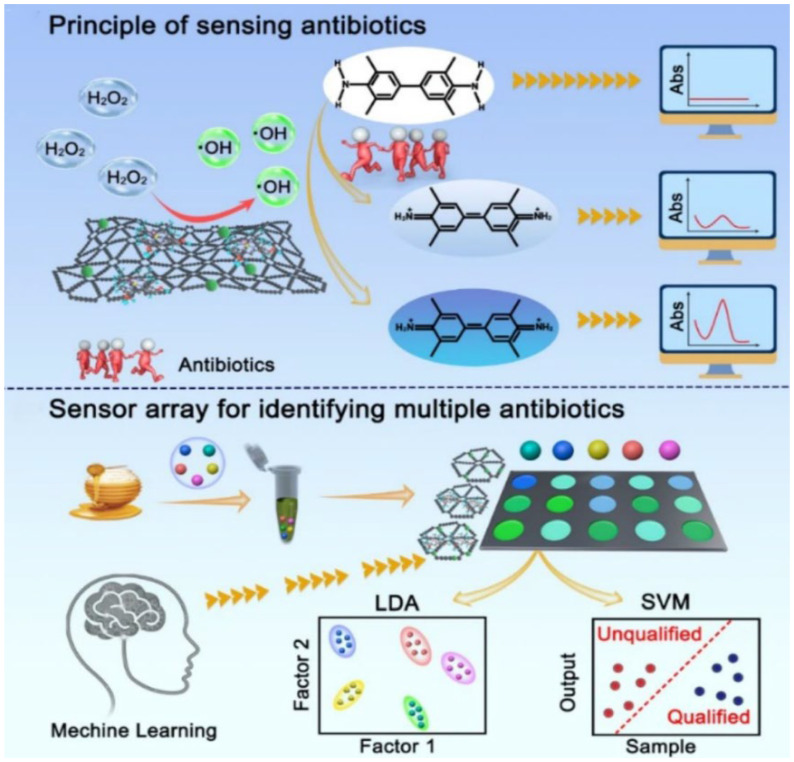
Working principle of POD-mimic GDY for sensing antibiotics and GDY-based sensor arrays assisted with machine learning for screening antibiotics (reprinted with permission from Ref. [80]. Copyright 2025, Elsevier).

For antibiotics, Qiu et al. reported the synthesis of a graphdiyne (GDY)-based dual-site POD-mimicking material, which comprised atomically loaded hemin molecules and Cu ions on GDY (GDY/Hemin/Cu), to develop sensor arrays [80]. In their study, interfacial interactions of hemin and Cu^2+^ with GDY were explored, and the POD-mimetic catalytic performance of the GDY/Hemin/Cu nanozyme was investigated. As illustrated in Figure 3, a three-channel color sensing array was fabricated using GDY/Hemin/Cu and the other two materials (GDY/Hemin and GDY/Cu). The array utilized TMB and H_2_O_2_ as enzymatic substrates, and antibiotics could compete with TMB to hinder the latter’s oxidation to result in a decrease in absorbance. The developed array demonstrated good selectivity, stability, and fast response to enable the accurate analysis of five antibiotics by LDA. Additionally, the SVM algorithm was utilized to establish a mathematical model to evaluate the target content.

### 4.2. Food Analysis

**Figure 4:**
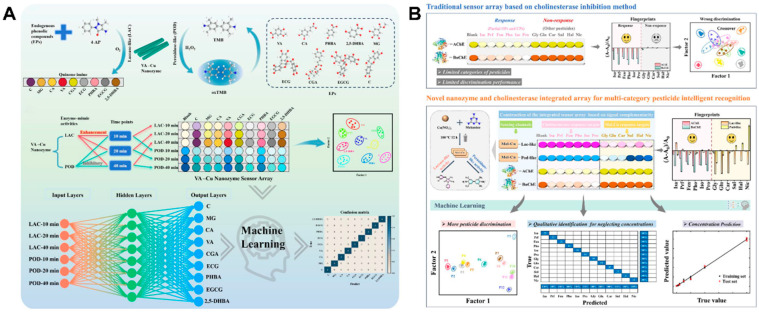
(**A**) Machine-learning-assisted nanozyme sensing array used in discriminating phenolic substances in food (reprinted with permission from Ref. [63]. Copyright 2024, American Chemical Society). (**B**) Machine-learning-enabled sensor array based on the integration of natural enzymes and nanozymes for the identification of multicategory pesticides (reprinted with permission from Ref. [64]. Copyright 2024, Elsevier).

On the one hand, nanozyme-based sensor arrays have found application potential in food nutrition analysis, including antioxidants [63,66,67,71], plant flavones [34], and unsaturated fatty acids [36]. For polyphenols with oxidation resistance, Liu’s group prepared a vanillic acid–Cu (VA-Cu) nanozyme with both POD- and LAC-mimicking activities [63]. The POD behavior of VA-Cu catalyzed TMB to produce blue oxTMB (Figure 4A). Because of the strong reducing ability of endogenous phenolic substances (EPs), the process was suppressed, and the degree of inhibition increased with increasing reaction time. In addition, the LAC behavior of the VA-Cu facilitated the oxidation of different EPs, leading to the generation of colored quinone imines. The catalytic degree also increased with increasing reaction time. According to the findings, a six-channel array (two enzyme-mimicking activities × three reaction times) was developed, which enabled the successful discriminant sensing of nine EPs. The authors also combined an ANN algorithm with the sensor array to achieve the accurate identification and prediction of EPs in black tea, honey, and grape juice.

For plant flavones, Qin et al. constructed a transition-metal-doped CeO_2_-based colorimetric array that was coupled with smartphone sensing [34]. The array was used to differentiate and analyze multi-ingredient flavonoids with the help of “segmentation-extraction-regression” deep learning (SER-DL). Mn-, Co-, and Fe-doped CeO_2_ nanozymes (CeMn, CeCo, and CeFe) with POD activity were prepared and utilized as sensing channels to design the sensor array based on the oxidation reaction of TMB under the catalysis of the nanozymes, which was competitively suppressed to different degrees by nobiletin, hesperidin, and tangeretin. Through the multihole parallel acquisition strategy of a self-developed mobile application (Quick Viewer), the RGB values of the color images were assessed. LDA was further conducted to separate the flavonoids at different levels, in mixtures with varying component ratios, and in citri reticulatae pericarpium (CRP) samples with different storage years. The SER-DL algorithm-assisted sensing array enabled the simultaneous quantification of the three flavonoids in CRP. The color features of single-hole images, which were segmented from array pictures, were extracted though a MobileNetV3-small algorithm to develop regression models. In the end, embedding the MobileNetV3-small algorithm into a smartphone to develop an application system (Intelligent Analysis Master) made the quantitative determination of flavonoids possible.

On the other hand, a few machine-learning-assisted nanozyme-based sensor arrays have been developed for the detection of food hazard factors such as pesticide residues [50,61,64,72] and bisphenol A [38]. For pesticide residues, Li’s group reported an integrated sensor array made of cholinesterase and a nanozyme, combined with machine learning classification and regression algorithms, to achieve the one-step qualitative recognition and quantitative analysis of a wide range of pesticides [64]. As shown in Figure 4B, a melamine–Cu nanozyme (Mel-Cu) was fabricated by a simple solvothermal synthetic process to present LAC and POD activities. The double catalytic activities of Mel-Cu were selected as two channels of the integrated sensing array, while other channels were inspired by the enzyme inhibition method and consisted of AChE and butyrylcholinesterase (BChE). The potential signaling complementarity of Mel-Cu and cholinesterase channels was favorable for enhancing pesticide species suitability. The ability of the fabricated array to identify multiple pesticides at various levels as well as pesticide mixtures was evaluated. To achieve the accurate prediction of unknown samples, a unified stepwise prediction model composed of a classification model neglecting concentrations and twelve regression models toward different pesticides was further constructed. As a result, the immunity and practical application feasibility of the fabricated sensing array were assessed and achieved satisfactory accuracy for blind samples. Recently, our group combined phosphatase-like nanozymes with sensor arrays together and constructed a kinetics-difference-driven organophosphorus hydrolase-mimicking nanozyme-coded pattern for the identification of *p*-nitrophenyl pesticides [48]. As the sole sensing unit, ultrasmall bare CeO_2_ nanoparticles were synthesized and exhibited favorable phosphatase-like activity. The designed CeO_2_ hydrolyzed *p*-nitrophenyl pesticides with different kinetics to *p*-nitrophenol, and reaction time was selected as the only variable to offer varying chromogenic signals for multiple analytes. After the color fingerprints were treated by pattern recognition including LDA and HCA, five commonly used *p*-nitrophenyl pesticides (methyl-paraoxon, paraoxon, methyl-parathion, parathion, and fenitrothion) were well identified and quantified. The designed pattern could identify different concentrations of individual *p*-nitrophenyl pesticides as well as their mixtures at various ratios. Significantly, our design showed three merits: first, direct hydrolysis of the analytes generated readable color signals, thus eliminating the requirement for additional enzymatic substrates and labels; second, the proposed pattern did not rely on redox reactions to catalyze targets, and it was free from the interference of redox substances in samples, which often caused potential impacts in previous oxidoreductase-based sensor arrays; third, by employing phosphatase-like bare nanoceria as the only sensing unit and reaction time as the sole variable, the developed sensor array showed the attractive features of facile construction, operation, and data processing.

### 4.3. Biomedical Sensing

**Figure 5:**
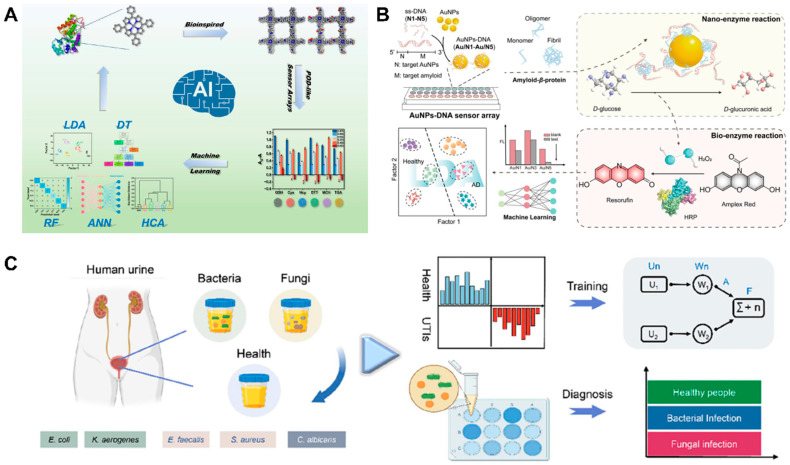
(**A**) Machine-learning-based nanozyme-involved sensor array for identifying and detecting biothiols (reused with the permission of Ref. [65], copyright@American Chemical Society). (**B**) Machine-learning-enabled bioenzyme/nanozyme integrated sensor array for detecting amyloids (reprinted with permission from Ref. [73]. Copyright 2023, American Chemical Society). (**C**) Nanozyme-involved colorimetric sensing array with the help of machine learning for the rapid and intelligent diagnosis of urinary tract infection (Reprinted with permission from Ref. [47]. Copyright 2024, American Chemical Society).

With their powerful function in high-throughput screening and identifying multiple targets in complex matrices, nanozyme-involved sensor arrays assisted by machine learning have also been intensively applied to detect small molecules such as biothiols [46,65,86], proteins [73,77,87,88], and pathogenic bacteria [37,47,89]. For biothiols, Lin’s group prepared two iron porphyrin covalent organic frameworks (Fe-COF-H and Fe-COF-OH) as nanozyme sensing units to fabricate a six-channel color sensing array of mercaptans (Figure 5A) [65]. Leveraging their outstanding POD catalytic activity, the obtained cross fingerprints were converted into an intuitive two-dimensional map using machine learning techniques such as LDA, decision tree (DT), RF, ANN, and HCA to identify multiple biothiols in complex systems accurately. To assess the practical feasibility of the sensor array, it was applied to differentiate cardiovascular disease patients by detecting varying levels of homocysteine in serum samples. It also distinguished different types of depilatory creams and identified normal and cancerous cells based on glutathione levels.

For proteins, Xu et al. employed the signal amplification impact of both nanozymes and bioenzymes to fabricate a fluorescence sensor array for the high-sensitivity identification of A*β*40 and A*β*42 peptides [73]. The system underwent a two-step coupling of nanozymes and bioenzymes to convert the weak interaction differences between the analytes and the sensing elements (Au NPs–DNA) into amplified fluorescence readout variations via adjusting the enzymatic activities (Figure 5B). The proposed strategy adsorbed ss-DNAs, an inhibitor of GOx-like Au NPs, onto the surface of Au NPs via noncovalent interactions and resulted in Au NP−DNA complexes with low catalytic activity. Upon the analytes were added, the catalytic activity increased or decreased according to the interactions between the Au NP−DNA complexes and A*β* peptides, resulting in the differential production of H_2_O_2_ from D-glucose. The generated H_2_O_2_ further oxidized Amplex red in the presence of horseradish peroxidase (HRP), generating fluorescence variations that were used to establish fingerprints of multiple A*β* peptides. By combining the strategy with machine learning algorithms, the hybrid nano-/biocatalyst sensor array achieved 100% accuracy in identifying several A*β* aggregates at a concentration of 200 nM. By taking advantage of the POD-like activity of g-C_3_N_4_ and its affinity to biomolecules, Qiu et al. constructed an array-based sensor for multiple protein assaying [87]. g-C_3_N_4_ catalyzed the chromogenic substrate TMB in the presence of H_2_O_2_ to produce a blue color. Proteins bearing various functional groups exhibited different affinities to the surface of g-C_3_N_4_, which might have influenced the interactions between g-C_3_N_4_ and the substrate. As a result, concomitant alterations in the catalytic efficiency of g-C_3_N_4_ occurred, leading to distinct initial velocities of catalytic reactions, times to reach reaction plateaus, and absorbance values of the final products. These parameters were reflected in the spectra of time-dependent absorbance changes as a result of the catalytic oxidation of TMB. By extracting the absorbance values at 652 nm within different reaction times, a fingerprint pattern was obtained. The collected fingerprints were treated by PCA to distinguish different proteins.

For pathogenic bacteria, Yang et al. presented the development of a machine-learning-assisted colorimetric nanoplatform based on an iron single-atom catalyst (Fe_1_−NC) with multiple nanozyme activities for the clinical diagnosis of urinary tract infection (UTI) [47]. In their study, a four-element colorimetric sensor array was built using the nanozyme with the help of four recognition ligands (antibiotics, boric acid, cetyltrimethylammonium bromide, and D-alanine acid); these foreign regulators were assembled on the surface of Fe_1_–NC to offer the fingerprints of pathogens (Figure 5C). After being processed by the uniform manifold approximation and projection (UMAP) algorithm, the obtained complex data pattern could be transformed to indicate the main UTI-related micro-organisms with varied order, genus, and species levels. Moreover, an SVM algorithm was further developed as a mathematical model to classify healthy people and patients with bacterial or/and fungal infections via urine analysis, offering a satisfactory diagnostic accuracy (as high as 97% for 60 clinical samples).

While the above studies demonstrate the potential of machine-learning-assisted nanozyme arrays for biomedical applications, significant challenges remain in sample matrix complexity, clinical validation, and physiological specificity. In biological fluids, abundant proteins such as albumin, immunoglobulins, electrolytes, and small molecules can interfere with nanozyme–target interactions. For example, in thiol assays, high levels of glutathione in serum may compete with target biothiols to bind to metal-based nanozymes such as Fe-COF-H, leading to false negative results [65]. This matrix effect was further exacerbated in diseased samples with altered metabolite profiles. To address this problem, surface functionalization of nanozymes, such as polyethylene glycol coating, has been used to reduce nonspecific adsorption, but optimization of different substrate types is still needed. Clinical validation is still limited in this case. Most work has relied on small sample cohorts or synthetic analogues, and multicenter, blinded validation is lacking compared with gold-standard methods. For example, while the Fe_1_–NC nanozyme array achieved 97% diagnostic accuracy in patients with UTI, its performance in populations with complex comorbidities, such as diabetes or renal disease, remains unproven. Physiological specificity is another obstacle. Nanozyme activity can vary dramatically in different physiological contexts, e.g., the acidic tumor microenvironment (pH 6.5) may enhance the peroxidase-like activity of MnO_2_ nanowires by two to three times compared with normal blood (pH 7.4) [38], potentially leading to false positives in cancer cell detection. In addition, endogenous reactive oxygen species (ROS) in tissues can interfere with redox-based nanozyme assays, so the development of ROS-insensitive nanozymes is needed. In addition, the long-term biocompatibility of nanozymes must be evaluated rigorously for in vivo applications, as unresolved toxicity issues may impede the progress of transformation. In the future, researchers are expected to break through the bottleneck of matrix complexity and clinical suitability and provide technical support for the development of nanozyme technology in biomedicine.

## 5. Conclusions

To sum up, this minireview summarizes the progress achieved to date in machine-learning-assisted nanozyme-based sensor arrays from the construction, empowerment, and application aspects. Nanozymes present unique advantages in activity regulation, high stability and large-scale preparation, and they have become promising alternatives to natural enzymes to provide catalytic amplified signals for biochemical detection and abundant optional variables in array sensing. Unlike the traditional “lock-and-key” paradigm, sensors arrays offer platforms that can achieve the high-throughput screening and analysis of multiple targets simultaneously, where machine learning algorithms help with complex signal collection, conversion, processing, and feedback in an intelligent way. The perfect combination of nanozymes, sensor arrays, and machine learning has enabled machine-learning-assisted nanozyme-based sensor arrays to be intensively used in identifying similar analytes in complicated matrices. It is believed that they will find wider use in environmental monitoring, food analysis, and biomedical detection.

**Figure 6:**
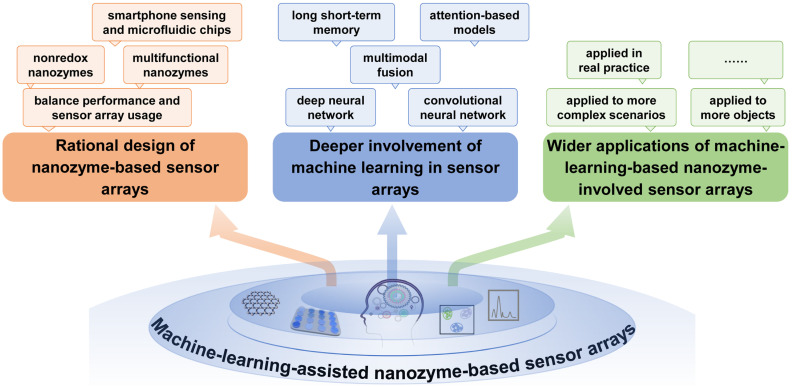
Illustration of challenges and future development trends in machine-learning-assisted nanozyme-based sensor arrays.

Although some breakthroughs have been made in research on nanozyme-based sensor arrays, some challenges and future trends should not be overlooked for their better development and practical applications, as illustrated in Figure 6.

(1) Rational design of nanozyme-based sensor arrays. The rational design of a sensor array is very important for its analytical performance, and more attention should be paid to it to advance sensor arrays. For example, nonredox nanozymes (such as hydrolase-mimicking activity) could be explored as sensing units to avoid the possible oxidation–reduction interference from sample matrices [48]. As nanozymes often possess some other interesting features apart from their catalytic function, developing multifunctional nanozymes (such as fluorescent nanozymes [60]) can provide multidimensional signals for more precise analysis. Of course, recognition performance should be ensured while simplifying the fabrication and use of sensor arrays as much as possible, thus reducing the material, operation, and data processing costs. Furthermore, combining a convenient and easy-to-use sensor array with smartphones and microfluidic chips may benefit fast and on-site data analysis and result presentation [34].
(2) Deeper involvement of machine learning in sensor arrays. Currently, only a limited number of machine learning algorithms have been incorporated into the data processing and result presentation of sensor arrays [90]. In fact, there are some other procedures that require the involvement of advanced algorithms to better facilitate performance improvement and convenient application. For example, machine learning can guide the iterative design and optimization of nanozyme materials with better catalytic properties [91,92], and machine learning can participate in signal pretreatment and noise suppression, as well as the screening and removal of false data. The comparison of different machine learning algorithms in the same scenario is also necessary to present effective, optimized and undistorted results. In addition, it is necessary to explore more advanced deep learning technologies to maximize the functionality of sensor arrays. For instance, CNN can be used to process image-like data generated by sensor arrays to achieve more accurate and efficient feature extraction; long short-term memory networks (LSTMs) can be used to analyze the dynamics of time series, which is crucial for real-time monitoring and predictive modeling; and the integration of multimodal data fusion technology can enhance the overall performance of sensor arrays by combining the information of different types of sensors. Future research can further explore the application of deep learning methods in nanozyme-based sensor arrays to develop more efficient, accurate and interpretable sensor systems.
(3) Wider applications of machine-learning-based nanozyme-involved sensor arrays. At present, nanozyme-based sensor arrays have found some laboratorial applications under the assistance of machine learning. Even so, their scope is expected to expand to more objects and more complex scenarios. For emerging analytes in specific fields, more types of receptors should be explored and combined with nanozymes to fabricate new types of sensor arrays. Furthermore, the use of machine-learning-assisted nanozyme-based sensor arrays in real practice is supposed. However, real-world deployment faces critical challenges. From a cost perspective, although nanozymes offer lower production costs than natural enzymes, scalable synthesis with consistent catalytic activity remains a bottleneck; for example, batch-to-batch variations in metal-doped carbon nanozymes can affect sensor reproducibility [69]. Device miniaturization, while advanced by smartphone and microfluidic integration [34,78], requires further innovation to compress multimodal sensing units (e.g., colorimetric–fluorescent–photothermal modules) into portable devices without compromising signal quality. Additionally, environmental factors (e.g., humidity, temperature) and matrix interference (e.g., proteins, salts in biological fluids) can alter nanozyme performance, necessitating robust calibration strategies. It is worth noting that regulatory approval is another hurdle: for biomedical applications, nanozyme-based sensors must meet strict biocompatibility criteria, but current guidelines on nanozyme characterization are fragmented, which complicates clinical validation. To address these, collaborative efforts across materials science, engineering, and regulatory science are urgent, including the establishment of nanozyme production standards and the development of self-calibrating sensor architectures.

## Figures and Tables

**Table 1:** Comparison of our work with previous related reviews.

Title	Nanozyme Activity and Sensor Array Construction	Machine Learning	Sensor Array Application	Ref.
Construction and Application of Nanozyme Sensor Arrays	① Types of nanozyme activities and their regulatory factors; ② Principles and methods of sensor array construction (colorimetric, ratio, fluorescence, multimodal)	Not mentioned	Small molecules, ions, pesticide residues, mycotoxin and bacteria, metal salts, phenolic compounds, antibiotics	[21]
Advances in the Application of Sensor Arrays Based on Nanozymes	① Classification and activity regulation of nanozymes; ② Sensor array construction strategies (multiunit signal integration, signal amplification and regulation)	Not involved	Small molecules, proteins, pesticide residues, metal ions	[22]
Machine-Learning-Assisted Nanozyme-Based Sensor Arrays: Construction, Empowerment, and Applications	① Nanozyme materials and catalytic activities; ② Core elements of sensor arrays, namely sensing variables (material difference, environmental regulation, time dependence, multiwavelength signal, multisubstrate response, multimicrosignal set) and signal outputs (single mode, multimode)	① Classical machine learning algorithms, including dimension reduction and classification (PCA, LDA, HCA) as well as prediction (SVW, ANN, RF); ② Deep learning and emerging algorithms, including CNN, GNN, and AM	Environmental detection, food analysis, biomedical sensing	This work

**Table 2:** Comparing the features of different sensing variables used in fabricating nanozyme-involved sensor arrays.

Sensing Variable	Feature	Advantage	Disadvantage	Applicable Scene
A series of materials with the same catalytic type	Unique fingerprints are obtained by taking advantage of the differences in catalytic activity of various nanozyme materials and their interactions with target substances	Obtaining unique fingerprints for a variety of targets	Increased material costs	Suitable for scenarios that require high specificity in recognizing multiple target objects
Reaction regulators for nanozyme catalysis	Sensor arrays are constructed by taking advantage of the sensitivity of nanozyme catalytic reactions to environmental conditions (such as pH)	Reducing material costs and obtaining different signals by adjusting reaction conditions	The regulators available are limited	Applicable to scenarios that are cost-sensitive and have a small number of target species categories
Reaction-time-dependent kinetic signals	Capturing signals generated at different time points during nanozyme catalytic reactions to construct sensor arrays	Simple operation, cost-effectiveness	Requiring precise time control and signal recording	Suitable for scenarios where reaction kinetics are distinct and targets affect kinetics differently
Signals recorded at different wavelengths	Using the absorbance differences of the same material at various wavelengths to construct sensor arrays	Providing additional signal dimensions	Requiring precise spectrometric measurement equipment	Suitable for scenarios where the material has significant absorbance differences at different wavelengths
Various substrates used in nanozyme catalysis	Employing different substrates as electron donors to participate in nanozyme reactions, offering distinct responses to similar analytes for identification	Obtaining different signals for multiple substrates	Requiring multiple substrates	Suitable for scenarios with substrate-selective nanozymes and multiple target analytes
Multiple activity types provided by the same material	Constructing sensor arrays by utilizing different catalytic types presented in the same nanozyme	Providing multiple reaction signals	Complex operation and requiring the recording of multiple signals	Suitable for scenarios with nanozymes having multiple catalytic activities
Multiple-dimensional signals provided by a multifunctional nanozyme	Constructing sensor arrays by combining the catalytic features of nanozymes with their electrical, optical, magnetic, and thermal properties	Offering multidimensional signals to enhance identification accuracy	High equipment requirements and complex signal processing	Suitable for scenarios requiring high-precision identification and multidimensional signal recording
Cross-use of more than one variable	Combining the above-mentioned sensing variables to achieve more precise sensing results	Providing a rich combination of signals to improve identification capability	Complex experimental design and data analysis	Suitable for complex scenarios requiring high precision and specificity to identify multiple targets

**Table 3:** Comparing the characteristics of different machine learning algorithms.

Machine Learning Algorithm	Data Type	Applicable Objective	Main Feature
PCA	Numerical data (high-dimensional data)	Dimensionality reduction, feature extraction	Unsupervised learning, linear transformation to project data into lower-dimensional space while retaining maximum variance
LDA	Numerical data (labeled)	Classification	Supervised learning, linear transformation to maximize class separation while minimizing within-class variance
HCA	Numerical data	Clustering	Unsupervised learning, builds a hierarchical tree structure to group data, suitable for exploratory data analysis
SVM	Numerical data (linearly or nonlinearly separable)	Classification, regression (SVR)	Finds the optimal hyperplane to maximize class margins, suitable for high-dimensional data and small sample problems
ANN	Numerical data	Classification, regression	Multilayer neural network structure that learns nonlinear relationships in data, suitable for complex pattern recognition
RF	Numerical data, categorical data	Classification, regression	Ensemble learning method based on decision trees, improving generalization through random sampling and feature selection
DNN	Numerical data (large-scale data)	Classification, regression	Multilayer neural network structure that learns complex nonlinear relationships but requires large amounts of data for training
CNN	Image data, sequential data	Classification, regression	Utilizes convolutional layers to extract local features, suitable for data with spatial or temporal local correlations
GNN	Graph-structured data (nodes and edges)	Classification, regression, node embedding	Models relationships between nodes, suitable for data with complex interactions
AM	Numerical data, sequential data	Classification, regression	Focuses on key parts of the data through attention mechanisms, enhancing model interpretability and performance
LSTMs	Sequential data (time series)	Classification, regression	Suitable for processing sequential data with long-term dependencies, capable of remembering and forgetting information

**Table 4:** Typical nanozyme-based sensor arrays assisted with machine learning for biochemical detection applications.

Nanozyme Material	Activity	Sensing Variable	Signal Mode	Machine Learning	Application	Ref.
VA-Cu	POD and LAC	Enzymatic activities and reaction times	Colorimetric	LDA, HCA, and ANN	Discrimination of phenolic compounds	[63]
Au NPs	GOx	Enzymatic substrates	Colorimetric	LDA, HCA, and DNN	Identification and discrimination of monosaccharides	[55]
Cu-BTC	LAC and POD	Enzymatic activities and multidimensional signals	Colorimetric and photothermal	LDA, HCA, and ANN	Intelligent recognition of antioxidant phenolic compounds	[66]
Fe-COF-H and Fe-COF-OH	POD	Signals at different wavelengths	Colorimetric	LDA, DT, ANN, HCA, and RF	Identification and detection of thiols	[65]
GMP-Cu and ASP-Cu	PPO	Nanozyme materials	Colorimetric	PLS-LDA, HCA, and BPNN	Identification of tea polyphenols and Chinese tea	[71]
Au NPs	GOx	ss-DNA regulators	Fluorescence	LDA	Detection of amyloids	[73]
CeCo, CeMn, and CeFe	POD	Nanozyme materials	Colorimetric	LDA and SER-DL	Discrimination and quantification of flavonoids	[34]
TPA@GQD	POD	Metal ion regulators	Colorimetric	LDA and HCA	Thiol discrimination and disease identification	[46]
MnO_2_, Ag-MnO_2_, Pd-MnO_2_, and Pt-MnO_2_	OXD	Nanozyme materials	Colorimetric	LDA and ISFE-DL	Detecting unsaturated fatty acids	[36]
Fe-N-C and Fe-N-C-Urea	OXD	Nanozyme materials	Colorimetric	LDA and HCA	Identification of oral cariogenic bacteria	[37]
GDY/Cu, GDY/Hemin, and GDY/Hemin/Cu	POD	Nanozyme materials	Colorimetric	LDA and SVM	Screening of multiple antibiotics	[80]
ASP-Cu	POD	Multidimensional signals	Colorimetric, fluorescence, and photothermal	LDA, HCA, and KNN	Identification of sulfonylurea pesticides	[61]
C_3_N_4_ NSs	POD	Aptamer regulators	Ratiometric fluorescence	LDA and HCA	Detection of exosomal proteins and cancer identification	[77]
CuO NPs	OXD	Signals at different wavelengths	Fluorescence	CNN-Max	Sugar identification	[74]
Mn-GY, Mn-GY-2N, and GY-2N	POD	Nanozyme materials	Colorimetric	HCA and LDA	Bisphenol identification	[38]

## Abbreviations

**Abbreviation**	**Full Name**
NPs	Nanoparticles
POD	Peroxidase
ML	Machine learning
OXD	Oxidase
CAT	Catalase
SOD	Superoxide dismutase
LAC	Laccase
TMB	3,3′,5,5′-Tetramethylbenzidine
PCA	Principal component analysis
LDA	Linear discriminant analysis
HCA	Hierarchical cluster analysis
SVM	Support vector machine
ANN	Artificial neural network
RF	Random forest
GOx	Glucose oxidase
OPD	o-Phenylenediamine
ABTS	2,2′-Azino-bis(3-ethylbenzthiazoline-6-sulfonic acid)
SERS	Surface enhanced Raman scattering
DAB	Diaminobenzidine
CNN	Convolutional neural network
KNN	K-nearest neighbor
PPO	Polyphenol oxidase
TCs	Tetracyclines
AChE	Acetylcholinesterase
BChE	Butyrylcholinesterase
DT	Decision tree
UTI	Urinary tract infection
ROS	Reactive oxygen species
MOFs	Metal–organic frameworks
SER-DL	Segmentation–extraction–regression deep learning
ISFE-DL	Image segmentation–feature extraction deep learning
PLS-DA	Partial least squares discriminant analysis
PLS-LDA	Partial least squares–linear discriminant analysis
UMAP	Uniform manifold approximation and projection
HRP	Horseradish peroxidase
DNN	Deep neural network
TC	Tetracycline
SVR	Support vector regression
GDY	Graphdiyne

## References

[1] Gao L., Zhuang J., Nie L., Zhang J., Zhang Y., Gu N., Wang T., Feng J., Yang D., Perrett S. (2007). Intrinsic Peroxidase-Like Activity of Ferromagnetic Nanoparticles. Nat. Nanotechnol..

[2] Wei H., Wang E. (2013). Nanomaterials with Enzyme-Like Characteristics (Nanozymes): Next-Generation Artificial Enzymes. Chem. Soc. Rev..

[3] Liang M., Yan X. (2019). Nanozymes: From New Concepts, Mechanisms, and Standards to Applications. Acc. Chem. Res..

[4] Huang Y., Ren J., Qu X. (2019). Nanozymes: Classification, Catalytic Mechanisms, Activity Regulation, and Applications. Chem. Rev..

[5] Wu J., Wang X., Wang Q., Lou Z., Li S., Zhu Y., Qin L., Wei H. (2019). Nanomaterials with Enzyme-Like Characteristics (Nanozymes): Next-Generation Artificial Enzymes (II). Chem. Soc. Rev..

[6] Li S., Zhang Y., Wang Q., Lin A., Wei H. (2022). Nanozyme-Enabled Analytical Chemistry. Anal. Chem..

[7] Diao Q., Chen X., Tang Z., Li S., Tian Q., Bu Z., Liu H., Liu J., Niu X. (2024). Nanozymes: Powerful Catalytic Materials for Environmental Pollutant Detection and Degradation. Environ. Sci. Nano.

[8] Zhang Y., Rui X., Simpson B.K. (2021). Trends in Nanozymes Development versus Traditional Enzymes in Food Science. Curr. Opin. Food Sci..

[9] Cao C., Yang N., Wang X., Shao J., Song X., Liang C., Wang W., Dong X. (2023). Biomedicine Meets Nanozyme Catalytic Chemistry. Coord. Chem. Rev..

[10] Jiang D., Ni D., Rosenkrans Z.T., Huang P., Yan X., Cai W. (2019). Nanozyme: New Horizons for Responsive Biomedical Applications. Chem. Soc. Rev..

[11] Zhang Y., Ya S., Huang J., Ju Y., Fang X., Ouyang X., Zeng Q., Zhou X., Yan X., Nie G. (2025). Spatial Isolation of Single Copper(I) Sites for Cascade Enzyme-Like Catalysis and Simultaneous Ferroptosis/Cuproptosis Boosted Immunotherapy. Exploration.

[12] Niu X., Cheng N., Ruan X., Du D., Lin Y. (2020). Review—Nanozyme-Based Immunosensors and Immunoassays: Recent Developments and Future Trends. J. Electrochem. Soc..

[13] Wang Q., Wei H., Zhang Z., Wang E., Dong S. (2018). Nanozyme: An Emerging Alternative to Natural Enzyme for Biosensing and Immunoassay. TrAC Trends Anal. Chem..

[14] Liang H., Chen X., Bu Z., Bai Q., Liu J., Tian Q., Tang Z., Li S., Diao Q., Niu X. (2024). When Nanozymes Meet Deoxyribonucleic Acid: Understanding Their Interactions and Biomedical Diagnosis Applications. Interdiscip. Med..

[15] Li X., Wang L., Du D., Ni L., Pan J., Niu X. (2019). Emerging Applications of Nanozymes in Environmental Analysis: Opportunities and Trends. TrAC Trends Anal. Chem..

[16] Zhang X., Wu D., Zhou X., Yu Y., Liu J., Hu N., Wang H., Li G., Wu Y. (2019). Recent Progress in the Construction of Nanozyme-Based Biosensors and Their Applications to Food Safety Assay. TrAC Trends Anal. Chem..

[17] Tian Q., Li S., Tang Z., Zhang Z., Du D., Zhang X., Niu X., Lin Y. (2024). Nanozyme-Enabled Biomedical Diagnosis: Advances, Trends, and Challenges. Adv. Healthc. Mater..

[18] Li T., Zhu X., Hai X., Bi S., Zhang X. (2023). Recent Progress in Sensor Arrays: From Construction Principles of Sensing Elements to Applications. ACS Sens..

[19] Rakow N.A., Suslick K.S. (2000). A Colorimetric Sensor Array for Odour Visualization. Nature.

[20] Askim J.R., Mahmoudi M., Suslick K.S. (2013). Optical Sensor Arrays for Chemical Sensing: The Optoelectronic Nose. Chem. Soc. Rev..

[21] Xia J., Li Z., Ding Y., Shah L.A., Zhao H., Ye D., Zhang J. (2024). Construction and Application of Nanozyme Sensor Arrays. Anal. Chem..

[22] Ma Y., Liu H., Li B., Lu N. (2024). Advances in the Application of Sensor Arrays Based on Nanozymes. Biosens. Bioelectron. X.

[23] Masson J.F. (2024). Roadmap for the Use of Machine Learning and Artificial Intelligence in Sensing. ACS Sens..

[24] Liu Q., Zhang A., Wang R., Zhang Q., Cui D. (2021). A Review on Metal- and Metal Oxide-Based Nanozymes: Properties, Mechanisms, and Applications. Nano-Micro Lett..

[25] Cai S., Yang R., Yan X. (2020). Noble Metal-Based Nanozymes. Nanozymology.

[26] Sun H., Zhou Y., Ren J., Qu X. (2018). Carbon Nanozymes: Enzymatic Properties, Catalytic Mechanism, and Applications. Angew. Chem. Int. Ed..

[27] Niu X., Li X., Lyu Z., Pan J., Ding S., Ruan X., Zhu W., Du D., Lin Y. (2020). Metal–organic Framework Based Nanozymes: Promising Materials for Biochemical Analysis. Chem. Commun..

[28] Fu R., Ma Z., Zhao H., Jin H., Tang Y., He T., Ding Y., Zhang J., Ye D. (2023). Research Progress in Iron-Based Nanozymes: Catalytic Mechanisms, Classification, and Biomedical Applications. Anal. Chem..

[29] Zhang H., Liang X., Han L., Li F. (2018). “Non-Naked” Gold with Glucose Oxidase-Like Activity: A Nanozyme for Tandem Catalysis. Small.

[30] Sun Y., Xu B., Pan X., Wang H., Wu Q., Li S., Jiang B., Liu H. (2023). Carbon-Based Nanozymes: Design, Catalytic Mechanism, and Bioapplication. Coord. Chem. Rev..

[31] Li S., Zhou Z., Tie Z., Wang B., Ye M., Du L., Cui R., Liu W., Wan C., Liu Q. (2022). Data-Informed Discovery of Hydrolytic Nanozymes. Nat. Commun..

[32] Wu Y., Chen W., Wang C., Xing D. (2023). Overview of Nanozymes with Phosphatase-Like Activity. Biosens. Bioelectron..

[33] Gabrielli L., Prins L.J., Rastrelli F., Mancin F., Scrimin P. (2020). Hydrolytic Nanozymes. Eur. J. Org. Chem..

[34] Qin Y., Zhong X., Liang C., Liang Z., Nong Y., Deng L., Guo Y., Li J., Zhang M., Tang S. (2024). Nanozyme-Based Colorimetric Sensor Arrays Coupling with Smartphone for discrimination and “Segmentation-Extraction-Regression” Deep Learning Assisted Quantification of Flavonoids. Biosens. Bioelectron..

[35] Shen L., Khan M.A., Wu X., Cai J., Lu T., Ning T., Liu Z., Lu W., Ye D., Zhao H. (2022). Fe–N–C Single-Atom Nanozymes Based Sensor Array for Dual Signal Selective Determination of Antioxidants. Biosens. Bioelectron..

[36] Zhong X., Qin Y., Liang C., Liang Z., Nong Y., Luo S., Guo Y., Yang Y., Wei L., Li J. (2024). Smartphone-Assisted Nanozyme Colorimetric Sensor Array Combined “Image Segmentation-Feature Extraction” Deep Learning for Detecting Unsaturated Fatty Acids. ACS Sens..

[37] Zhang Y., Khan M.A., Yu Z., Yang W., Zhao H., Ye D., Chen X., Zhang J. (2024). The Identification of Oral Cariogenic Bacteria through Colorimetric Sensor Array Based on Single-Atom Nanozymes. Small.

[38] Xia J., Fu R., Li Z., Ding Y., Zhao H., Ye D. (2025). Graphyne-Supported Manganese Single-Atom Nanozyme Sensor Array for Bisphenol Identification. Talanta.

[39] Zhu Y., Wu J., Han L., Wang X., Li W., Guo H., Wei H. (2020). Nanozyme Sensor Arrays Based on Heteroatom-Doped Graphene for Detecting Pesticides. Anal. Chem..

[40] Xiao Y., Gong W., Zhao M., Zhang M., Lu N. (2023). Surface-Engineered Prussian Blue Nanozymes as Artificial Receptors for Universal Pattern Recognition of Metal Ions and Proteins. Sens. Actuators B Chem..

[41] Wang X., Qin L., Zhou M., Lou Z., Wei H. (2018). Nanozyme Sensor Arrays for Detecting Versatile Analytes from Small Molecules to Proteins and Cells. Anal. Chem..

[42] Wu F., Wang H., Lv J., Shi X., Wu L., Niu X. (2023). Colorimetric Sensor Array Based on Au2Pt Nanozymes for Antioxidant Nutrition Quality Evaluation in Food. Biosens. Bioelectron..

[43] Liu B., Xue Y., Gao Z., Tang K., Wang G., Chen Z., Zuo X. (2021). Antioxidant Identification Using a Colorimetric Sensor Array Based on Co-N-C Nanozyme. Colloids Surf. B.

[44] Li Y., Liu Y., Zhang Y., Dong M., Cao L., Jiang K. (2024). A Simple Ag–MoS2 Hybrid Nanozyme-Based Sensor Array for Colorimetric Identification of Biothiols and Cancer Cells. RSC Adv..

[45] Wang H., Wu F., Wu L., Guan J., Niu X. (2023). Nanozyme Colorimetric Sensor Array Based on Monatomic Cobalt for the Discrimination of Sulfur-Containing Metal Salts. J. Hazard. Mater..

[46] Zhu X., Li T., Hai X., Bi S. (2022). A nanozyme-based colorimetric sensor array as electronic tongue for Thiols Discrimination and Disease Identification. Biosens. Bioelectron..

[47] Yang J., Li G., Chen S., Su X., Xu D., Zhai Y., Liu Y., Hu G., Guo C., Yang H.B. (2024). Machine Learning-Assistant Colorimetric Sensor Arrays for Intelligent and Rapid Diagnosis of Urinary Tract Infection. ACS Sens..

[48] Li S., Chen X., Tian Q., Liu J., Tang Z., Niu X. (2025). Kinetics Difference-Driven Organophosphorus Hydrolase-Like Nanozyme-Coded Pattern for Identifying p-Nitrophenyl Pesticides. Anal. Chem..

[49] Chen X., Wu Y., Zeng Y., Liu J., Niu X. (2025). Single-Nanozyme Single-Readout Enabled Efficient Identification of Polyphenols for Chinese Tea Authentication and Brewing Evaluation. Food Chem..

[50] Bai Y., Nie S., Gao W., Li N., Zhu P., Zhang L., Yu J. (2024). Enzyme-Nanozyme Cascade Flow Reactor Synergy with Deep Learning for Differentiation and Point-of-Care Testing of Multiple Organophosphorus Pesticides. Adv. Funct. Mater..

[51] Li F., Jiang J., Peng H., Li C., Li B., He J. (2022). Platinum Nanozyme Catalyzed Multichannel Colorimetric Sensor Array for Identification and Detection of Pesticides. Sens. Actuators B Chem..

[52] Zeng K., Wang Y., Tao Y., Huang R., Dong Q., Deng C., Wu Q., Niu X., Wei D., Zhang Z. (2025). Single Sensing Element with Multiple Adsorption Peaks Assembled Nanozyme Sensor Array for Discrimination of Multiple Metal Ions. Microchem. J..

[53] Jing W., Qiang S., Jia Z., Shi Q.H., Meng X., Yu M., Ma H., Zhao K., Dai Y. (2023). Smartphone-Integrated Nanozymes Sensor Array for High Throughput Recognition of Organophosphorus Pesticides. Sens. Actuators B Chem..

[54] Yue N., Lai Y., Wu J., Zhang Q., Qi W., Su R. (2024). Optimization of Metal–Organic Framework Nanozyme Activity via Histidine Modification for Simultaneous Pesticide Detection. Chem. Eng. J..

[55] Huang S., Xiang H., Lv J., Zhu D., Yu L., Guo Y., Xu L. (2024). Au Nanozyme-Based Colorimetric Sensor Array Integrates Machine Learning to Identify and Discriminate Monosaccharides. J. Colloid Interface Sci..

[56] Niu X., Liu B., Hu P., Zhu H., Wang M. (2022). Nanozymes with Multiple Activities: Prospects in Analytical Sensing. Biosensors.

[57] Chen Z., Li S., Guan Y., Wu C., Qian Y., Zhou H., Qian Y., Yue Y., Yue W. (2024). Ultrasmall CuMn-His Nanozymes with Multienzyme Activity at Neutral pH: Construction of a Colorimetric Sensing Array for Biothiol Detection and Disease Identification. ACS Appl. Mater. Interfaces.

[58] Song D., Lei L., Tian T., Yang X., Wang L., Li Y., Huang H. (2023). A Novel Strategy for Identification of Pesticides in Different Categories by Concentration-Independent Model Based on a Nanozyme with Multienzyme-Like Activities. Biosens. Bioelectron..

[59] Wu J., Li S., Wei H. (2018). Multifunctional Nanozymes: Enzyme-Like Catalytic Activity Combined with Magnetism and Surface Plasmon Resonance. Nanoscale Horiz..

[60] Diao Q., Tang Z., Liu J., Niu X. (2025). Fluorescent Nanozymes: Emerging Versatile Materials Advancing Analytical Chemistry. TrAC Trends Anal. Chem..

[61] Tian T., Song D., Zhen L., Bi Z., Zhang L., Huang H., Li Y. (2025). Colorimetric–Fluorescence–Photothermal Tri-Mode Sensor Array Combining the Machine Learning Method for the Selective Identification of Sulfonylurea Pesticides. Biosens. Bioelectron..

[62] Huang Y., Gong L., Xie C., Qin W., Wang M., Hu L., Xia Z. (2025). Anchoring Biomimetic Zn Site in Metal–Organic Framework Nanozyme to Enhance Phosphatase-Like Catalytic Activity for Discrimination of Organophosphorus Pesticides. Chem. Eng. J..

[63] Jing W., Yang Y., Shi Q., Wang Y., Liu F. (2024). Machine Learning-Based Nanozyme Sensor Array as an Electronic Tongue for the Discrimination of Endogenous Phenolic Compounds in Food. Anal. Chem..

[64] Song D., Zou Y., Tian T., Ma Y., Huang H., Li Y. (2024). Machine Learning-Assisted Melamine-Cu Nanozyme and Cholinesterase Integrated Array for Multi-Category Pesticide Intelligent Recognition. Biosens. Bioelectron..

[65] Hu C., Xie W., Liu J., Zhang Y., Sun Y., Cai Z., Lin Z. (2024). Bioinspired Iron Porphyrin Covalent Organic Frameworks-Based Nanozymes Sensor Array: Machine Learning-Assisted Identification and Detection of Thiols. ACS Appl. Mater. Interfaces.

[66] Xu J., Wang Y., Li Z., Liu F., Jing W. (2025). Machine Learning Assisted Multi-Signal Nanozyme Sensor Array for the Antioxidant Phenolic Compounds Intelligent Recognition. Food Chem..

[67] Yang X., Zou B., Zhang X., Yang J., Bi Z., Huang H., Li Y. (2024). A Sensor Array Based on a Nanozyme with Polyphenol Oxidase Activity for the Identification of Tea Polyphenols and Chinese Green Tea. Biosens. Bioelectron..

[68] Tian T., Song D., Zhang L., Huang H., Li Y. (2024). Facile and Selective Recognition of Sulfonylurea Pesticides Based on the Multienzyme-Like Activities Enhancement of Nanozymes Combining Sensor Array. J. Hazard. Mater..

[69] Ren E., Qiu H., Yu Z., Cao M., Sohail M., Lu G.P., Zhang X., Lin Y. (2024). Nanozyme Sensor Array Based on Fe, Se Co-Doped Carbon Material for the Discrimination of Sulfur-Containing Compounds. J. Hazard. Mater..

[70] Li X., Li L., Tang H., Xie C., Zhao Y., Wu P. (2025). Non-Colorimetric Sensing with 3,3′,5,5′-Tetramethylbenzidine. Sens. Actuators B Chem..

[71] Yang X., Bi Z., Yin C., Huang H., Li Y. (2024). A Novel Hybrid Sensor Array Based on the Polyphenol Oxidase and Its Nanozymes Combined with the Machine Learning Based Dual Output Model to Identify Tea Polyphenols and Chinese Teas. Talanta.

[72] Zeng X., Chen H., Long F., She X., Lan W., Long W., Wang S., Wei L., She Y., Fu H. (2025). Cloud Machine Learning-Enhanced Peroxidase-Like Enzyme Visual Sensor for Rapid Detection of Sulfur-Containing Pesticides. Sens. Actuators B Chem..

[73] Xu Y., Qian C., Yu Y., Yang S., Shi F., Xu L., Gao X., Liu Y., Huang H., Stewart C. (2023). Machine Learning-Assisted Nanoenzyme/Bioenzyme Dual-Coupled Array for Rapid Detection of Amyloids. Anal. Chem..

[74] Noreldeen H.A.A., He S.B., Wu G.W., Peng H.P., Deng H.H., Chen W. (2024). Deep Convolutional Neural Network-Based 3D Fluorescence Sensor Array for Sugar Identification in Serum Based on the Oxidase-Mimicking Property of CuO Nanoparticles. Talanta.

[75] Wang X., Qin L., Lin M., Xing H., Wei H. (2019). Fluorescent Graphitic Carbon Nitride-Based Nanozymes with Peroxidase-Like Activities for Ratiometric Biosensing. Anal. Chem..

[76] Lan C., Meng L., Xu N. (2023). Dual-Channel Ratiometric Colorimetric Sensor Array for Quantifcation and Discrimination of o-, m-, and p-Phenols. Food Anal. Methods.

[77] Liu M.X., Zhang H., Zhang X.W., Chen S., Yu Y.L., Wang J.H. (2021). Nanozyme Sensor Array Plus Solvent-Mediated Signal Amplification Strategy for Ultrasensitive Ratiometric Fluorescence Detection of Exosomal Proteins and Cancer Identification. Anal. Chem..

[78] Wang L., Chen Y., Ji Y., Wang L., Liu X., Wang F., Li C. (2024). Nanozyme-Inhibited SERS Multichannel Paper-Based Sensor Array for the Quantification and Identification of Biothiols and Cancer Cells Based on Three Ag-Based Nanomaterials. Anal. Chem..

[79] Song D., Tian T., Wang L., Zou Y., Zhao L., Xiao J., Huang H., Li Y. (2024). Multi-Signal Sensor Array Based on a Fluorescent Nanozyme for Broad-Spectrum Screening of Pesticides. Chem. Eng. J..

[80] Qiu X., Yang J., Bai R., Zhao M., Shao C., Zhao Q., Gu Y., Guo C., Li C.M. (2025). Dual-Site Peroxidase-Mimic Graphdiyne-Based Colorimetric Sensor Arrays with Machine Learning for Screening of Multiple Antibiotics. Sens. Actuators B Chem..

[81] Zhang Y., Wang M., Shao C., Liu T., Sun M., Wu C., Su G., Wang Y., Ye J., Hu H. (2024). Nanozyme-Induced Deep Learning-Assisted Smartphone Integrated Colorimetric and Fluorometric Dual-Mode for Detection of Tetracycline Analogs. Anal. Chim. Acta.

[82] Jiang S., Su G., Wu J., Song C., Lu Z., Wu C., Wang Y., Wang P., He M., Zhao Y. (2023). Co_3_O_4_/CoFe_2_O_4_ Hollow Nanocube Multifunctional Nanozyme with Oxygen Vacancies for Deep-Learning-Assisted Smartphone Biosensing and Organic Pollutant Degradation. ACS Appl. Mater. Interfaces.

[83] Wang L., Xu X., Liu P., Wang M., Niu X., Pan J. (2021). A single-nanozyme colorimetric array based on Target-Induced Differential Surface Passivation for Quantification and Discrimination of Cl^−^, Br^−^ and I^−^ Ions. Anal. Chim. Acta.

[84] Wu S., Guo D., Xu X., Pan J., Niu X. (2020). Colorimetric Quantification and Discrimination of Phenolic Pollutants Based on Peroxidase-Like Fe_3_O_4_ Nanoparticles. Sens. Actuators B Chem..

[85] Xu X., Wu S., Guo D., Niu X. (2020). Construction of a Recyclable Oxidase-Mimicking Fe_3_O_4_@MnOx-Based Colorimetric Sensor Array for Quantifying and Identifying Chlorophenols. Anal. Chim. Acta.

[86] Zhou X., Li L., Wang Y., Kong T., Cao Z., Xie H., Liang W., Wang Y., Qian S., Chao J. (2023). Nanozyme Inhibited Sensor Array for Biothiol Detection and Disease Discrimination Based on Metal Ion-Doped Carbon Dots. Anal. Chem..

[87] Qiu H., Pu F., Ran X., Liu C., Ren J., Qu X. (2018). Nanozyme as Artificial Receptor with Multiple Readouts for Pattern Recognition. Anal. Chem..

[88] Wang L., Hu Z., Wu S., Pan J., Xu X., Niu X. (2020). A Peroxidase-Mimicking Zr-Based MOF Colorimetric Sensing Array to Quantify and Discriminate Phosphorylated Proteins. Anal. Chim. Acta.

[89] Lu Z., Lu N., Xiao Y., Zhang Y., Tang Z., Zhang M. (2022). Metal-Nanoparticle-Supported Nanozyme-Based Colorimetric Sensor Array for Precise Identification of Proteins and Oral Bacteria. ACS Appl. Mater. Interfaces.

[90] Chen X., Liu J., Tang Z., Liu S., Peng J., Liang H., Niu X. (2025). Machine Learning-Enabled Time-Resolved Nanozyme-Encoded Recognition of Endogenous Mercaptans for Disease Diagnosis. Anal. Chem..

[91] Wei Y., Wu J., Wu Y., Liu H., Meng F., Liu Q., Midgley A.C., Zhang X., Qi T., Kang H. (2022). Prediction and Design of Nanozymes using Explainable Machine Learning. Adv. Mater..

[92] Gao Y., Zhu Z., Chen Z., Guo M., Zhang Y., Wang L., Zhu Z. (2024). Machine Learning in Nanozymes: From Design to Application. Biomater. Sci..

